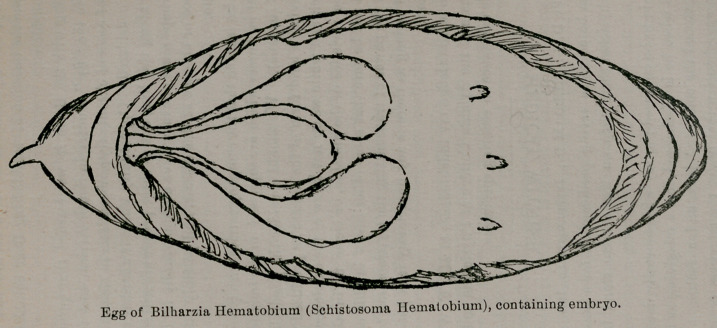# Belharzia Hematobium; Report of Seven Cases

**Published:** 1905-11

**Authors:** Claude A. Smith

**Affiliations:** Atlanta, Ga.


### BILHARZIA HEMATOBIUM; REPORT OF SEVEN CASES.

BY CLAUDE A. SMITH, M.D., ATLANTA.

Upon seeing the company of the Boer War Spectacle parading the streets of Atlanta in March last, it occurred to the writer that possibly they might be infected with the parasite known as bilharzia hematobium, or schistosoma hematobium, as this parasite is very common in the Transvaal. Through the courtesy of Dr. S. G. C. Pinckney, of Atlanta, and of Captain Hyndon, of the Boer con-contingent, specimens of urine were obtained from forty-one Boers and from four of the South African negroes.

While desirous of obtaining specimens from every member of the company, both Boers and English (more than three hundred in all), in order to determine the exact number of cases of the disease, if any might be present, yet as the company was in Atlanta less than three days, and as their various duties kept them quite busy most of the time, it was not possible to obtain more than the forty-five specimens. Also, it was impossible to obtain a detailed history of each case, as time did not permit. All of the specimens were centrifugated, and microscopical examination made. Seven of the specimens were found to contain the eggs of the bilharzia hematobium, one of the cases being among the negroes, and the other six cases being among the Boer soldiers.

When Captain Hyndon was first spoken to regarding the disease, he stated that no doubt many of the men had had the disease in the past, as the symptoms were common in the Transvaal, but he felt sure that none had the disease at the present time. Also, in talking with the soldiers none seemed to think that they were affected with the disease at this time, but almost all gave a history indicating that they had contracted it at some time in the past. Many of the

English soldiers also gave histories indicating that they had contracted the disease, as many of them had lived in the Transvaal for a number of years. However, no specimens were obtained from them.

All of the cases were apparently chronic cases, as microscopic inspection of the specimens did not indicate the presence of any blood or blood clots in the urine. Also, the amount of infection was not great, as the men were apparently not inconvenienced or affected by the presence of the disease—frequent micturition being the only abnormality. In three of the specimens the eggs were fairly numerous upon centrifugation, in the others they were somewhat scant. Microscopic examination showed a few red corpuscles in each specimen, and two of the specimens contained quite a number of pus cells. No tube casts were found in any of the specimens. Some of the eggs appear to be prematurely discharged, being sometimes as small as one-half the size of the adult egg, and not containing an embryo, but instead containing a few granules or globules without any definite arrangement. Others show further increase in size, and contain definitely arranged masses of granules, and close inspection with a one-sixth objective showed a "boiling" motility in some of these masses.

From the fact that seven cases of the disease were found in forty-five persons examined, and the history of the others indicated that the disease existed among them in as great a proportion, it would appear that there might be a considerable number of cases of the disease among the others of this company from South Africa. And, while there is some uncertainty as to just how this parasite gets into the human body, whether by food or drinking water or by means of the skin, we know that the history of the cases and the development of the larvas indicate that it finds its intermediate host in the fresh river water. Whether this intermediate host belongs to the crustacea, as reported by some, or other organism, it is well for us to consider the possibility of contaminating our streams with this parasite, especially in view of the fact that we have no remedy for the disease should it once gain a foothold in this country. This is of importance, not only on account of the number of cases among these Boers who are at present in

Egg of Bilharzia Hematobium (Schistosoma Hematobium), containing embryo.

this country, but especially so in view of the fact that at the present time the question of establishing Boer colonies in Georgia is being considered.

In talking with the Boers it was learned that quite a number had been in the "fly" country in South Africa, and therefore the possibity of cases of chronic trypanosomiasis in this same company should be considered. Opportunity was not afforded to make observations along this line, but as these people are still in the United States, perhaps some one may have an opportunity of determining the presence or absence of this disease, and possibly other diseases among them.

It was also learned that the company carries with it some "salted" horses, that is, horses which are supposed to be immune to trypanosomiasis (nagana). We should consider the possibility of these animals having a mild form of the disease as produced by trypanosoma brucei, and while we do not know of the existence of any one of the seven varieties of the glossina, or tsetse fly in this country, yet we can not be sure that some other insect in this country does not possess the power of transmitting the trypanosoma to cattle and horses, and as long as there is any uncertainty it at least calls for consideration.

I wish to acknowledge my indebtedness to Mr. F. W. Schnauss, undergraduate of the Atlanta College of Physicians and Surgeons, for valuable assistance in securing specimens.

## Figures and Tables

**Figure f1:**